# Antenatal Syphilis Screening Using Point-Of-Care Testing in Low- and Middle-Income Countries in Asia and Latin America: A Cost-Effectiveness Analysis

**DOI:** 10.1371/journal.pone.0127379

**Published:** 2015-05-26

**Authors:** Andreas Kuznik, Christine Muhumuza, Henry Komakech, Elsa M. R. Marques, Mohammed Lamorde

**Affiliations:** 1 Infectious Diseases Institute, Makerere College of Health Sciences, Kampala, Uganda; 2 Celgene Corporation, Summit, New Jersey, United States of America; 3 School of Public Health, Makerere College of Health Sciences, Kampala, Uganda; 4 School of Social and Community Medicine, University of Bristol, Bristol, United Kingdom; Seattle Childrens Hospital, UNITED STATES

## Abstract

**Background:**

Untreated syphilis in pregnancy is associated with adverse clinical outcomes to the infant. In low- and middle-income countries in Asia and Latin America, 20%-30% of women are not tested for syphilis during pregnancy. We evaluated the cost-effectiveness of increasing the coverage for antenatal syphilis screening in 11 Asian and 20 Latin American countries, using a point-of-care immunochromatographic strip (ICS) test.

**Methods:**

The decision analytical cost-effectiveness models reported incremental costs per disability-adjusted life years (DALYs) averted from the perspectives of the national health care payer. Clinical outcomes were stillbirths, neonatal deaths, and congenital syphilis. DALYs were computed using WHO disability weights. Costs included the ICS test, three injections of benzathine penicillin, and nurse wages. Country-specific inputs included the antenatal prevalence of syphilis and the proportion of women in the antenatal care setting that are screened for syphilis infection as reported in the 2014 WHO baseline report on global sexually transmitted infection surveillance. Country-specific data on the annual number of live births, proportion of women with at least one antenatal care visit, and per capita gross national income were also included in the model.

**Results:**

The incremental cost/DALY averted of syphilis screening is US$53 (range: US$10-US$332; Prob<1*per capita GDP=99.71%) in Asia and US$60 (range: US$5-US$225; Prob<1*per capita GDP=99.77%) in Latin America. Universal screening may reduce the annual number of stillbirths by 20,344 and 4,270, neonatal deaths by 8,201 and 1,721, cases of congenital syphilis by 10,952 and 2,298, and avert 925,039 and 197,454 DALYs in the aggregate Asian and Latin American panel, respectively.

**Conclusion:**

Antenatal syphilis screening is highly cost-effective in all the 11 Asian and 20 Latin American countries assessed. Our findings support the decision to expand syphilis screening in countries with currently low screening rates or continue national syphilis screening programs in countries with high rates.

## Introduction

Congenital syphilis remains a major infectious cause of morbidity and mortality in neonates, infants and children in resource limited settings[1]. Untreated maternal syphilis in pregnancy is associated with adverse pregnancy outcomes and clinical outcomes to the infant. This is documented in a recent meta-analysis, which found that 53.4%–81.8% of women with untreated syphilis had adverse outcomes, compared to 10.2%–20.8% of women without syphilis [2]. In low- and middle-income countries in Asia and Latin America, roughly one out of three women are not tested for syphilis during pregnancy, with wide variations from country to country [3]. Neonates who survive with congenital syphilis are at risk of low birth weight, premature delivery, congenital anomalies, active syphilis in the infant, and longer-term sequelae, including deafness and neurologic impairment [4,5].

Testing and treating syphilis in pregnant women has been shown to decrease stillbirths, perinatal deaths, and incidence of congenital syphilis [6,7]. Syphilis testing is often recommended, but costs associated with screening programs are barriers to implementation in lower income countries. Recently introduced immunochromatographic strip (ICS) tests are rapid point-of-care treponemal tests that have sensitivity and specificity estimates comparable to those of laboratory-based syphilis screening tests [8,9]. In this study, we sought to determine the cost-effectiveness of expanding the screening for antenatal syphilis using point-of-care ICS tests in 31 low and middle-income countries in Asia and Latin America where data on syphilis prevalence were available.

## Methods

### Overview

The structure of the decision analytic model was adapted from a recent cost-effectiveness analysis of ICS testing and subsequent treatment relative to no testing and no treatment from the national health care payer perspective across 43 countries in sub-Saharan Africa (SSA) and is published elsewhere [10]. Our analysis applied the same model structure to the specific epidemiologic and economic context of 11 populous low- and middle-income countries in Asia and 20 low-and middle-income countries in Latin America. The current syphilis screening rate was available for 9 out of 11 countries in Asia and all 20 countries in Central and South America. For the 2 Asian countries (Bangladesh and Laos) where this rate was not available, we used the regional weighted average rate of screening among countries with available data in the respective region.

Results were displayed in terms of the incremental cost-effectiveness ratio (ICER). We also conducted probabilistic sensitivity analyses (PSA) by randomly selecting from 14 key model inputs (See Tables 1 and 2) and repeating this process 10,000 times, thus generating a 95% confidence interval around the base case ICER, as well as an estimate of the likelihood that the ICER fell below World Health Organization (WHO) recommended cost-effectiveness thresholds [11]. Furthermore, we report a syphilis prevalence target rate below which it would no longer be cost-effective to conduct routine screenings in the antenatal care population and compare how the current syphilis prevalence rate compares to the target rate. All analyseswere performed in Excel 2007 (Microsoft Corporation, Redmond, WA, USA) and TreeAge Pro 2014 (Treeage Software Inc., Williamstown, MA, USA).

**Table 1:** General Model Inputs.

Model Input	Basecase	95% CI	Distribution	Reference
ICS Test Sensitivity	86.0%	74.5% -94.1%	Beta	Tucker et al.[22]
ICS Test Specificity	99.0%	97.8%–99.7%	Beta	Tucker et al.[22]
Stillbirth, Untreated Mother with Syphilis	25.6%	17.8%–33.4%	Beta	Gomez et al.[2]
Neonatal Mortality, Untreated Mother with Syphilis	12.3%	9.1%–15.9%	Beta	Gomez et al.[2]
Congenital Syphilis, Untreated Mother with Syphilis	15.5%	7.1%–26.2%	Beta	Gomez et al.[2]
Penicillin Effectiveness in Reducing Stillbirths, RR	82.0%	67.0%–90.0%	Log-Normal	Blencowe et al.[6]
Penicillin Effectiveness in Reducing Neonatal Mortality, RR	80.0%	68.0%–87.0%	Log-Normal	Blencowe et al.[6]
Penicillin Effectiveness in Reducing Congenital Syphilis, RR	97.0%	93.0%–98.0%	Log-Normal	Blencowe et al.[6]
Congenital Syphilis Disability Weight	0.315	0.159–0.471	Normal	Murray and Lopez[23]
Discount Rate	3.0%	1.4%–5.3%	Beta	WHO[11]^a^

^a^ Reference [13] suggests a range of 0%-6% for one way sensitivity analyses, but does not report a suggested 95% confidence interval for probabilistic sensitivity analyses.

**Table 2:** Country Specific model inputs.

Country	Syphilis prevalence[3]	Women receiving antenatal syphilis screening [3]	Number of live births[24]	Antenatal care coverage [24]	Stillbirth rate [12]	Neonatal mortality rate [13]	Hourly nurse wage in dollars [15]
**Asia**							
Bangladesh	0.6%	68.6%^a^	3,016,000	49.8%	3.6%	2.6%	$1.02
Cambodia	0.1%	49.3%	317,000	89.1%	1.8%	1.9%	$0.55[16]^b^
China	0.2%	95.1%	16,364,000	94.1%	1.0%	0.9%	$2.48
India	0.6%	69.2%	27,098,000	75.1%	2.2%	3.2%	$2.40[25]
Indonesia	1.2%	0.1%	4,331,000	93.3%	1.5%	1.5%	$0.85
Laos	0.8%	68.6%^a^	140,000	71.0%	1.4%	1.8%	$0.51[26]
Malaysia	0.1%	99.5%	579,000	83.4%	0.6%	0.3%	$8.12[27]
Myanmar	0.6%	11.8%	824,000	83.1%	2.0%	3.0%	$0.24
Philippines	0.1%	33.0%[28]	2,358,000	91.1%	1.6%	1.2%	$1.34
Thailand	0.1%	91.6%	824,000	99.1%	0.4%	0.8%	$5.62
Vietnam	0.1%	27.0%[29]	1,458,000	93.7%	1.3%	1.5%	$0.53[30]
**Latin America**							
Argentina	1.1%	90.9%	693,000	99.0%	0.5%	1.0%	$4.07
Belize	0.5%	92.8%	8,000	94.0%	1.2%	0.8%	$6.97
Bolivia	1.3%	58.0%	264,000	86.0%	1.7%	2.2%	$2.47
Brazil	0.8%	88.2%	2,996,000	98.0%	1.0%	1.3%	$6.76
Chile	0.2%	100.0%	245,000	95.0%	0.9%	0.5%	$4.48
Colombia	1.0%	73.6%	910,000	97.0%	0.6%	1.1%	$3.63
Costa Rica	0.3%	87.0%	73,000	90.0%	0.5%	0.6%	$5.02
Cuba	0.1%	100.0%	110,000	100.0%	0.8%	0.3%	$2.50
Dominican Republic	3.4%	13.7%	216,000	99.0%	1.2%	1.2%	$2.51
Ecuador	0.1%	67.8%	298,000	84.0%	1.2%	1.0%	$4.69[16]^c^
El Salvador	0.2%	89.6%	126,000	94.0%	1.4%	0.6%	$2.93
Guatemala	0.4%	50.9%	473,000	93.0%	1.0%	1.5%	$2.21
Haiti	3.9%	68.4%	260,296	84.0%	1.5%	0.8%	$4.91[14]
Honduras	0.1%	40.7%	205,000	92.0%	1.8%	1.1%	$1.73
Mexico	0.2%	82.0%	2,195,000	96.0%	0.5%	0.7%	$3.83[16]^b^
Nicaragua	0.2%	70.9%	138,000	90.0%	1.4%	1.3%	$0.66
Paraguay	2.1%	60.5%	158,000	96.0%	1.9%	1.3%	$3.73[16]^b^
Peru	0.5%	78.5%	591,000	95.0%	1.0%	0.9%	$2.82
Uruguay	1.5%	82.6%	49,000	96.0%	0.9%	0.5%	$8.06
Venezuela	1.9%	96.1%	598,000	94.0%	1.1%	0.8%	$4.40

^a^Used continental average as proxy

^b^Based on code ILO-N: Health and Social Work

^c^Based on code ILO-851: Human Health Activities

### Epidemiology and Health Outcomes

The prevalence of syphilis infection in the antenatal care setting is reported in the 2014 WHO baseline report on global sexually transmitted infection surveillance [3] and ranged from a low of 0.1% (Cambodia, Malaysia, Philippines, Thailand and Vietnam) to a high of 1.2% (Indonesia) among the 11 Asian countries and ranged from a low of 0.1% (Cuba, Ecuador and Honduras) to a high of 3.9% (Haiti) in Latin America. Among untreated syphilis-infected women, the rates of stillbirth, early neonatal death, and congenital syphilis have been previously reported at 25.6%, 12.3% and 15.5%, respectively [2], and we assumed a constant risk of these events across all countries in our panel. Across the 31 countries in the model, we applied these three estimates to seropositive women in the “No screen and test” branch, as well as women with a false negative test result in the “Screen and test branch”. We used estimates from the literature for the average risk of stillbirth, neonatal death, and congenital syphilis to fall by 82%, 80%, and 97%, respectively, after treatment [6]. In our model, the risk of stillbirth was assumed to fall to 4.6% (i.e. 25.6% reduced by 82%), the risk of neonatal death to 2.5% (i.e. 12.3% reduced by 80%), and the risk of congenital syphilis to 0.5% (i.e. 15.5% reduced by 97%) among women with a true positive ICS test result that subsequently received penicillin therapy. For the true negative and false positive endpoints in our model, we assumed a risk of congenital syphilis equal to zero, and country-specific rates of stillbirth and early neonatal mortality [12,13]. Tables 1 and 2 report the general and country-specific epidemiological inputs.

### Direct Medical Costs

Patients in the intervention arm incurred the cost of the ICS test plus health care staff costs to administer and interpret the test. Patients with a positive test result further incurred the cost of one benzathine penicillin injection immediately following the positive result, two additional injections at follow-up visits, and staff costs of administering the three injections. Costs were expressed in 2012 US dollars. ICS tests were valued at $0.74 and penicillin injections at $1.92 for all countries [10]. Staff time to deliver the ICS test was estimated 17.5 minutes and 20 minutes for each injection treatment [14]. Occupational Wages around the World (OWW) data was available for 6 Asian and 16 Latin Americancountries in the model. Staff time was valued using recently reported hourly wage estimates for code 154 (Professional nurse, general), or, if not available, code 61 (occupational health nurse). Wages were inflated to 2012 price levels using local consumer price indices, and converted to US$ at 2014 exchange rates [15]. Hourly wage estimates for the remaining 9 countries, was retrieved from the International Labour Organization database of labour statistics, when available [16], or the literature, otherwise (Table 2). Since all relevant costs were accrued at the point of care and no future cost-offsets were assumed, cost estimates were not discounted.

## Results

Model results are displayed in Table 3. The annual number of live births in the two regions of interest is approximately 57.3 million and 10.6 million in the Asian and Latin American cohorts, respectively. Among these, 82.3% in Asia (47.2 million live births) and 95.5% in Latin America (10.1 million live births) report at least one antenatal care visit. The proportion of women screened for syphilis during pregnancy in these two regions is estimated at 68.6% and 81.5%, respectively. This suggests that approximately 14.8 million women in Asia and 1.9 million women in Latin America are not screened for syphilis during their antenatal care visit. However, the size of the population that is not screened is unevenly distributed within the two regions. Among Asian countries the largest numbers are estimated for India (6.3million unscreened), Indonesia (4.0 million), the Philippines (1.4 million), and Vietnam (1.0 million); while Mexico (0.4 million) and Brazil (0.3 million) account for the majority of unscreened pregnancies in the Latin American region. In contrast, Malaysia, Chile, and Cuba report syphilis screening rates approaching 100% in their respective populations.

**Table 3:** Model Results.

Country	Stillbirth averted	NND Averted	Congenital Syphilis Averted	DALY’s Averted	Increase in Direct Medical Costs in US Dollars	Costs/DALY Averted	Probability Screening is Cost Effective	Prevalence Target Rate	Current/Target Prevalence Rate
**Asia**									
Bangladesh	594	240	320	27,220	$537,764	$20	99.78%	0.023%	26
Cambodia	30	12	16	1,338	$138,744	$104	99.75%	0.019%	5
China	317	128	171	14,748	$1,176,868	$80	99.78%	0.005%	40
India	7,895	3,183	4,250	354,981	$9,798,213	$28	99.68%	0.018%	33
Indonesia	10,169	4,099	5,474	465,814	$4,527,004	$10	99.81%	0.006%	200
Laos	52	21	28	2,380	$31,037	$13	99.83%	0.014%	57
Malaysia	1	0	0	24	$7,868	$332	99.74%	0.006%	17
Myanmar	761	307	409	34,204	$543,912	$16	99.81%	0.017%	35
Philippines	302	122	163	13,840	$1,738,427	$126	99.79%	0.009%	11
Thailand	14	6	8	673	$171,664	$255	99.82%	0.009%	11
Vietnam	209	84	113	9,818	$960,217	$98	99.68%	0.012%	8
**Sum/weighted average**	**20,344**	**8,201**	**10,952**	**925,039**	**$19,631,720**	**$53**	**99.71%**	**0.013%**	**37**
**Latin America**									
Argentina	144	58	78	6,785	$132,188	$19	99.81%	0.003%	367
Belize	1	<1	<1	27	$1,600	$60	99.80%	0.012%	42
Bolivia	260	105	140	11,868	$155,780	$13	99.69%	0.012%	108
Brazil	582	235	313	27,088	$1,012,350	$37	99.74%	0.004%	200
Chile	0	0	0	5	$504	$109	99.78%	0.003%	67
Colombia	489	197	263	22,859	$459,649	$20	99.78%	0.005%	200
Costa Rica	5	2	3	256	$19,981	$78	99.76%	0.005%	60
Cuba	<1	<1	<1	1	$171	$156	99.80%	0.004%	25
Dominican Republic	1,317	531	709	61,321	$331,032	$5	99.81%	0.005%	680
Ecuador	17	7	9	796	$179,043	$225	99.76%	0.008%	13
El Salvador	5	2	3	240	$20,894	$87	99.74%	0.008%	25
Guatemala	181	73	98	8,378	$322,117	$38	99.82%	0.008%	50
Haiti	566	228	305	25,037	$181,887	$7	99.71%	0.055%	71
Honduras	23	9	13	1,093	$148,283	$136	99.62%	0.011%	9
Mexico	159	64	86	7,519	$746,942	$99	99.74%	0.003%	67
Nicaragua	15	6	8	709	$36,417	$51	99.78%	0.014%	14
Paraguay	264	106	142	12,249	$125,352	$10	99.86%	0.010%	210
Peru	127	51	68	5,921	$203,371	$34	99.86%	0.005%	100
Uruguay	26	10	14	1,217	$27,872	$23	99.77%	0.004%	375
Venezuela	87	35	47	4,086	$50,181	$12	99.85%	0.003%	633
**Sum/weighted average**	**4,270**	**1,721**	**2,298**	**197,454**	**$4,155,616**	**$60**	**99.77%**	**0.006%**	**139**

The incremental direct medical cost associated with increasing screening rates from current levels to universal screening for all 47.2 million and 10.1 million women that receive antenatal care in Asia and Latin America is estimated at $19.6 million and $4.2 million, respectively, with a breakdown of the budget impact per country reported in Table 3. The aggregate annual number of adverse birth events that could potentially be avoided by universal syphilis screening and subsequent penicillin treatment in the antenatal care settings in Asia is estimated as follows—stillbirth: 20,344, neonatal death: 8,201, congenital syphilis: 10,952, resulting in an estimated 925,039 DALYs that could be averted. The corresponding estimates for the Latin American region are—stillbirth: 4,270, neonatal death: 1,721, congenital syphilis: 2,298, resulting in an estimated 197,454 DALYs. The largest annual number of DALYs that could potentially be averted through universal screening is estimated for Indonesia (465,814), and India (354,981).

The weighted average cost/DALY averted is estimated at $53 in Asia and $60 in Latin America, respectively, and ranges from $10 (Indonesia) to $332 (Malaysia) and $5(Dominican Republic) to $225 (Ecuador) (Table 3). Probabilistic sensitivity analyses results are robust to simultaneous variation of all model parameters. The number of iterations for the 31 countries that resulted in highly cost-effective ICERs (falling below a given country’s GDP per capita) exceeded 99%. Syphilis screening would remain highly cost-effective at syphilis prevalence rates of around 0.013% (or 13 cases per 100,000 pregnancies) in Asia, and 0.006% (or 6 cases per 100,000 pregnancies) in Latin America. Syphilis prevalence rates could fall, on average, 37 times in Asia and 139 in Latin America relative to currently observed levels before universal syphilis screening during antenatal care ceases to be cost-effective. In 10 out of the 31 countries included in the analysis, the current prevalence exceeds the target rate by a factor of 100 or more.

## Discussion

Our study assessed the economic value associated with screening and subsequent treatment for active syphilis infection in the antenatal care settings of 31 low- and middle-income countries in Asia and Latin America. Our findings suggest that syphilis screening is highly cost-effective in the 31 countries assessed, using WHO thresholds of cost-effectiveness [11], with a probability of over 99% for all countries.

In Latin America and Asia, approximately 7–8 out of 10 women are screened for syphilis during pregnancy. This rate is higher rate than sub-Saharan African countries, where less than 40% of eligible women are screened [10], but considerably lower than European countries, where screening rates are close to 100%, despite low incidence rates (generally below 0.2%) [3]. The public health burden associated with antenatal syphilis infection in Latin America also exceeds that of European countries. The congenital syphilis rate per 100,000 live births in Europe ranges from 0.0 in Denmark, Greece, Hungary, Ireland, Italy, and Norway to 0.6 in Germany, 8.3 in Poland, and 24.5 in Portugal [3]; compared to 57.3 in Peru, 109.6 in Argentina, 151.6 in Uruguay, and 311.6 in Brazil, in our panel. Countries in the Asian region do not routinely report the congenital syphilis rate per 100,000 live births [3].

Recent WHO guidance on the elimination of mother-to-child transmission of HIV and syphilis, established targets of antenatal care coverage of 95%, and syphilis screening of 95%, in order to achieve a goal of fewer than 50 cases of congenital syphilis per 100,000 live births [17]. Among the 31 countries included in our analysis, only Chile and Cuba currently meet both of the WHO targets, leaving over 20 million live births in the remaining 29 countries not screened for syphilis infection during pregnancy. One possible explanation is that syphilis infection may not be recognized as a national health priority in countries where prevalence rates are low. In Honduras, for example, the prevalence rate is reported at 0.1% (100 per 100,000 pregnancies), and less than half of all pregnant women are screened. Our findings suggest that routine screening is highly cost-effective even in countries with relatively low prevalence, and that syphilis prevalence rates would have to fall to much lower levels (e.g. below 11 cases per 100,000 pregnancies in Honduras) before antenatal syphilis screening is no longer cost-effective. Our results are primarily driven by the assumption of the availability of a low-cost, point-of-care ICS test as the screening tool, and by the availability of a highly effective but low-cost treatment option for syphilis (benzathine penicillin). The screening and treatment costs would have to be substantially higher for our model to display less cost-effective results.

Our study identified countries in the two regions in which syphilis screening programs may be particularly appealing. In Asia, this would include Indonesia, for example, which reports a syphilis prevalence rate of 1.2%, a high rate of access to antenatal care at 93.3%, but only 0.1% of pregnant women screened for syphilis. Indonesia alone accounts for almost half of the DALY burden in our panel of Asian countries and at this prevalence rate, the cost/DALY averted is estimated at merely $10. Latin American countries, such as the Dominican Republic and Haiti, report a relatively high prevalence rate of 3.4% and 3.9%, respectively, which translate into a cost per DALY averted of $5 and $7, respectively, suggesting that the screening rates in these two high prevalence countries should be increased from current levels of about 14% and 69%.

Practical strategies to increase the rate of syphilis screening in antenatal care may involve integrating rapid syphilis testing into existing HIV screening services. Recent evidence indicates that such an integrated approach was feasible in resource-limited settings and resulted in increased screening rates in South Africa [18], as well as Uganda and Zambia [19], and was found to be highly cost-effective in China [20]. Eventually, it could be expanded further to also include screening for Hepatitis B in a triple point-of-care test [21].

Our study is not without limitations. As with any cost-effectiveness model, ours is only as accurate as the published model inputs that feed into it; inaccurately reported data inputs would translate into biased model results. In two countries, we were not able to assess the current proportion of pregnant women that are screened for syphilis infection, and had to rely on the regional average as a proxy. Although our ICER calculations are not sensitive to this parameter, our estimates of the DALY burden and potential budget impact associated with increased screening rates may prove to be incorrect. The WHO infection surveillance report on country specific syphilis prevalence rates [3] does not specify what type of syphilis screening test was used to generate local prevalence data, so we are not able to confirm whether the reported seroprevalence data actually consisted of cases of active syphilis infection. Furthermore, our results are specific to the ICS test and may not be generalizable to other diagnostic methods that may be in use in various countries. Our estimated country-specific average hourly nurse wages ignores potential regional wage variations within in a country and assumes that additional hours of nursing time are available at this marginal cost. Nurse wages may increase with greater demand and other structural costs of increasing health care provision were not available for inclusion in the model. The costs of training, outreach and shipping of tests necessary to scale up antenatal syphilis screenings across the two regions were not formally included in our analysis, which probably underestimates the true economic cost of increasing screening rates. Last, we assume that all with a positive ICS test result receive three injections of penicillin, however, patient attrition may occur at follow-up visits, which could reduce the effectiveness and potentially also the cost-effectiveness of penicillin therapy.

In conclusion, antenatal syphilis screening is highly cost-effective in all 31 low and middle-income countries in Asia and Latin America that were assessed. Our findings support the decision to expand syphilis screening in countries with currently low prevalence rates of syphilis and to continue national syphilis screening programs in countries with high rates.
